# Prognostic gene expression assays in breast cancer: are two better than one?

**DOI:** 10.1038/s41523-018-0063-9

**Published:** 2018-05-22

**Authors:** Joseph A. Sparano

**Affiliations:** Montefiore Medical Center, Albert Einstein College of Medicine, Bronx, NY USA

Gene expression assays are commonly used to aid clinical decision-making in early stage estrogen receptor (ER)-positive, Her2-negative breast cancer because they provide complementary prognostic information to clinicopathologic features. Currently available assays include the 21-gene Oncotype DX® Recurrence Score (Genomic Health, Inc., Redwood City, CA),^1^ the 70-gene MammaPrint® assay (Agendia Inc., USA),^2^ and others.^3^ The 21-gene and 70-gene assays also provide predictive information for chemotherapy benefit^4,5^ or lack thereof because of a very low recurrence risk with endocrine therapy alone,^2,6,7^ providing a foundation for their recommendation in practice guidelines.^3,8^ Evidence supporting the clinical utility of the 70-gene assay was provided by the MINDACT trial, which showed that tumors associated with high clinical risk and low genomic risk by the 70-gene assay had a distant metastasis-free survival (DMFS) of 95% with endocrine therapy alone, which met the prespecified primary trial end point.^2^ Information provided by these assays result in a treatment change for up to 25%, usually in the direction of sparing chemotherapy.^9^

Among the currently available assays, the 21-gene assay was one of the first to become commercially available and shown to be predictive of chemotherapy benefit,^4,5^ factors contributing to its widespread use.^10^ The 21-gene assay provides a continuous recurrence score (RS) that allows more precise estimation of recurrence risk than a categorical classification, and also a categorical classification that includes not only “low-risk” (RS <18) and “high-risk” categories (RS >30), but also an “intermediate-risk” category (RS 18–30). Alternative RS cutpoints were proposed to define low risk (RS <11) and high risk (RS >25)^11^ to reflect an exclusively Her2-negative population, and reanalysis of the B20 validation study demonstrated a 2% 10-year DMFS rate for those with a RS <11 treated with tamoxifen alone, and similar chemotherapy benefit using the lower RS cutpoint of 26 or higher (10-year DMFS rate 63% versus 88%, hazard ratio [HR]: 0.29, *p* < 0.0001)^11^ as the original higher RS cutpoint of 31 or higher (10-year DMFS 61% versus 88%, HR: 0.26, *p* < 0.001).^4^ The Trial Assigning Individualized Options for Treatment (TAILORx) was designed to prospectively determine whether adjuvant hormonal therapy is not inferior to chemohormonal therapy alone in patients with a “mid-range” RS of 11–25 (who had a 10-year DMFS rate of 5% with tamoxifen alone in the B20 trial), and to confirm the excellent outcomes for those with a RS <11 treated with hormone therapy alone. Although results from the low-risk arm have confirmed expectations,^6^ results from the “mid-range” group are still awaited. It is noteworthy that 67% of TAILORx participants had a RS of 11–25 compared with only 43% of B20 subjects, reflecting clinicians selectively ordering the test in clinical situations, where there is therapeutic equipoise.^11^ Identifying the optimal management strategy for this group remains a major unmet clinical need, for both node-negative and node-positive disease.

In order to address this need, Tsai et al. described a prospective trial in which the impact of the 70-gene assay to guide treatment decisions was evaluated in 840 patients early stage ER-positive breast cancer with an “intermediate” RS of 18–30.^12^ Chemotherapy was removed from the treatment recommendation in 29% with a “low-risk” signature (45% of group), and added in 37% of those with a “high-risk” signature (55% of group). Is this a reasonable approach to pursue in clinical practice outside the context of this trial?

The answer is no, for a number of reasons. First, although reports have indicated only a moderate degree of concordance in risk classification by 21-gene assay and 70-gene assay,^13^ there is no information about the analytic validity, or association with actual clinical outcomes, of their combined use. The report by Tsai et al. is no exception, since it focused on how adding the 70-gene assay impacts adjuvant chemotherapy use, not actual clinical benefit resulting from a treatment change (i.e., clinical utility). Second, it is fairly obvious that application of a binary test result (“low-risk” vs. “high-risk”) to a group characterized as “intermediate-risk” by the 21-gene assay, however defined, will reclassify that group into two separate risk groups and influence clinical recommendations—an experiment with a predictable outcome. Third, this approach results in added assay costs without a clear return on investment, as the proportion of patients receiving chemotherapy actually increased by 8% with application of the 70-gene assay, and without much impact on patients with a RS of 26–30, few of whom were re-classified as “low-risk” by the 70-gene assay. Lastly and most importantly, the report by Tsai et al. provides no information regarding clinical risk as defined in MINDACT, which classified node-negative, ER-positive, Her2-negative disease as clinically low risk if the primary tumor was ≤3 cm and associated with low histologic grade, ≤2 cm if intermediate grade, or ≤1 cm if high grade;^2^ given that 87% had node-negative disease, 76% had tumors ≤2 cm, and 79% had low-intermediate grade tumors, it is likely that most had clinically low-risk disease as defined in MINDACT. Among the 478 patients in MINDACT who had clinically low risk, genomically high-risk disease, there was no significant benefit from chemotherapy (5-year DMFS 94% vs. 96%, HR: 0.90, *p* = 0.80), although the trial was underpowered for this comparison.

Taken together, these considerations call into question the major conclusion of the authors that use the 70-gene assay in this setting “…provides clinically actionable information…”, as there is little evidence that the actions taken would result in clinical benefit. Using any of the other currently available gene expression assays, which are also driven largely by genes reflecting ER signaling and proliferation,^14^ is not likely to be a fruitful approach when there is an “intermediate” RS, however defined. Other approaches that merit further evaluation include refining the 21-gene assay by adding other highly prognostic genes,^15,16^ or using RS in combination with other assays that reflect other biologic processes.^17^
